# What Matters and What Matters Most for Survival After age 80? A Multidisciplinary Exploration Based on Twin Data

**DOI:** 10.3389/fpsyg.2021.723027

**Published:** 2021-09-22

**Authors:** Boo Johansson, Valgeir Thorvaldsson

**Affiliations:** Department of Psychology and Centre for Ageing and Health (AgeCap), University of Gothenburg, Gothenburg, Sweden

**Keywords:** predictors, survival, longevity, oldest-old adults, self-assessments

## Abstract

Given research and public interest for conditions related to an extended lifespan, we addressed the questions of what matters and what matters most for subsequent survival past age 80. The data was drawn from the population-based and multidisciplinary Swedish OCTO Twin Study, in which a sample (*N* = 699) consisting of identical and same-sex fraternal twin pairs, followed from age 80 until death, provided detailed data on health, physical functioning, life style, personality, and sociodemographic conditions. Information concerning date of birth and death were obtained from population census register. We estimated heritability using an ACE model and evaluated the role of multiple predictors for the mortality-related hazard rate using Cox regression. Our findings confirmed a low heritability of 12%. As expected, longer survival was associated with being a female, an apolipoprotein E (APOE) e4 allele non-carrier, and a non-smoker. Several diseases were found to be associated with shorter survival (cerebrovascular, dementia, Parkinson’s, and diabetes) as well as certain health conditions (high diastolic blood pressure, low body mass index, and hip fracture). Stronger grip and better lung function, as well as better vision (but not hearing), and better cognitive function (self-evaluated and measured) was related to longer survival. Social embeddedness, better self-evaluated health, and life-satisfaction were also significantly associated with longer survival. After controlling for the impact of comorbidity, functional markers, and personality-related predictors, we found that sex, cerebrovascular diseases, compromised cognitive functioning, self-related health, and life-satisfaction remained as strong predictors. Cancer was only associated with the mortality hazard when accounting for other co-morbidities. The survival estimates were mostly in anticipated directions and contained effect sizes within the expected range. Noteworthy, we found that some of the so-called “soft-markers” remained strong predictors, despite a control for other factors. For example, self-evaluation of health and ratings of life-satisfaction provide additional and valuable information.

## Introduction

What make humans survive into late life and what are the lessons from those who survive into advanced ages (i.e., nowadays often defined as beyond 80 years of age)? Many answers are proposed, although often based on anecdotal evidence and several questions remain unanswered. Researchers typically recognize that this complex question presents major empirical challenges (see e.g., Slagboom et al., 2011; Olshansky and Carnes, 2019). In a review, Johansson and Bjälkebring (2015), also pointed to the lack of an overarching theory of longevity, and that survival into old age is determined by multiple genetic and environmental factors that interact in a complex manner over the entire life span. As example, we may expect that already at the embryonic level, individuals may experience health influences that later affect their likelihood for longevity. It is therefore difficult to capture all dimensions of survival into a single theory. However, we can at least identify factors that are more or less strongly associated with survival and longevity in older ages, which provide empirical contributions to the current fragmented theory building.

There is a lack of studies simultaneously evaluating and comparing the effect sizes of multiple indicators of subsequent survival, especially in very old individuals. Thus, the rationale for the present study was to use a representative population-based twin sample, aged 80 and older, to explore the relative importance of a broad set of potential candidate variables (i.e., genetic information, socio- demographics, life style, personality characteristics, markers of health, and well-being) for their role in subsequent survival. Over the life-course we can assume that the importance of some predictors increase while others decrease, dependent on birth-cohort and population characteristics. Furthermore, we know relatively little about the role of heritability for subsequent survival and predictors for longevity in the oldest population segment.

In an early account, Palmore (1969) summarized findings from a longitudinal study of people in the age range from 60 to 94 years. He analyzed an extensive list of potential candidates, including health indicators, intelligence, engagement in various activities, attitudes and wellbeing markers, adjustment and happiness ratings, socio-economic status and conditions, and parents’ age at death. Based on a longevity quotient (LQ), determined as the observed number of years lived after examination, divided by the expected number of years remaining for persons of a given sex, age, and race. Palmore concluded that the most important factors for longevity were: (a) to maintain a sense of usefulness and satisfying role in society; (b) to maintain a positive view of life; (c) to maintain good physical functioning; and (d) to avoid smoking.

Many empirical studies, following Palmore (1969), emphasize that multiple interacting factors contribute to late life survival. This becomes obvious in studies of the substantial longevity increase observed in many populations across the globe (e.g., Sadana et al., 2013). We can attribute increased survival to beneficial living conditions, from improved early life exposures to better late life living standards and treatments that act to preserve health, vitality, and overall functioning. The role of multiple factors for an increased survival becomes obvious also when we make comparisons across birth cohorts of older adults. In that respect, we typically find that number of years in school is associated with a longer life, a finding that led Lundborg et al. (2016) to conclude that education may work as a crucial vehicle to improve public health and thereby survival.

The role of genetics has long been a major focus in studies of longevity and survival into advanced ages (e.g., Kallmann and Sander, 1948) and several twin studies have been conducted to determine such influences. These studies typically converge on findings that approximately 20–30% of the between-person variation in life span can be attributed to genetic influences. Genetic influences are small before age 60, but increase thereafter (Christensen et al., 2006). Herskind et al. (1996), as example, estimated the heritability of longevity to be 0.26 for men and 0.23 for women. Interestingly, the heritability estimates were largely constant across the three examined birth cohorts in this study (i.e., with cohorts born between 1870 and 1900). Ljungquist et al. (1998) analyzed survival data in a sample of 3,656 identical and 6,849 same-sex fraternal twin pairs, born 1886 to 1900, and showed that more than one third of the variance could be attributed to genetic influences. They found that the remaining variance was entirely due to non-shared environmental factors. Most studies converge at the conclusion that longevity is only moderately heritable, although the estimates may differ depending on various population characteristics, examined age span, and other selection criteria.

As expected, there is limited evidence for the existence of a single longevity gene, since our genes tend to interact. However, among the many potential gene variants, the ε*4* allele of the apolipoprotein E (APOE) is typically identified to carry an elevated mortality risk (Christensen et al., 2006; Nebel et al., 2011). This is mainly because of the role of this variant in metabolic mechanisms leading to reduction in old age brain integrity, and further to compromised cognitive health and functioning (Jacobsen et al., 2010). In the early 90’s, telomere length was proposed as an informative biomarker and predictor of the remaining lifespan (see e.g., Haussmann and Mauck, 2008). Telomeres are dynamic chromosome-end structures that guard the genomic stability. Based on twin comparisons, Bakaysa et al. (2007) demonstrated that telomere length, in a sample aged 63–95 years, acted as a reliable biomarker of subsequent survival over a 7-year period, after controlling for genetic resemblance. Intra-pair analyses revealed that those with the shortest telomeres exhibited a three times’ greater risk of dying during the follow-up period.

Diseases and compromised health are prevalent in old age (e.g., Morewitz and Goldstein, 2007). In a previous report, we showed that among members of a cohort of hardy survivors, from a pre-welfare society (at focus also in the present study), only five individuals out of 702 (i.e., 0.7%) were free of any disease (Nilsson et al., 2002). In that report, we presented data for 44 common diseases, uniformly classified according to ICD-10, based on a concurrent review of medical records, self-reports, and registration of marker drugs used to determine and confirm a diagnosis. Our findings demonstrated the prevalence of morbidity and health problems among the oldest-old. The mean number of diagnoses in the entire sample was 6.7 with a range from 0 to 18. We found that 18% of the sample had ten or more diagnoses, while only 9% had two or less. Thus, an accurate portrayal of health status in late life needs to account for multi-morbidity and its role in restricting survival (see Nilsson et al., 2002)^1^.

Among the many disease affecting survival in late life, we typically find eight common causes of death. The list first encompasses heart and blood vessel diseases, including heart failures (^∗^57%), cardiac infarct (^∗^20%), and arrhythmias (^∗^10%), conditions associated with high blood pressure (^∗^57%). The second major life shortening disease category is cancer (^∗^25%), including breast cancer, colon cancer, and skin cancer, and malignant blood and bone marrow diseases that cause leukemia. Third, is chronic obstructive pulmonary disease (^∗^COPD and asthma, 13%), including chronic bronchitis and emphysema, that compromises breathing and oxygenation, and where smoking is a major risk factor. Fourth, cerebrovascular disease or stroke/CVD (^∗^24%), which restricts the blood flow to the brain or causes brain damage through hemorrhage and with significant risk factors like diabetes, hyperlipidemia, and smoking. A fifth disease category is dementia (^∗^20%), including Alzheimer’s disease, characterized by cognitive decline with progressive memory loss, personality changes, and finally a more complete loss of overall function associated with a significantly increased mortality risk (e.g., Johansson and Zarit, 1997; Wetterberg et al., 2021). A sixth category is represented by diabetes (^∗^17%), a metabolic disease that also affects the immune system with an increased risk of stroke, heart disease, and other circulatory problems. A seventh category is pneumonia and influenza, which often affect older adults with an already compromised health [currently seen in the coronavirus (COVID-19) pandemic]. Eight, falls and other accidents, leading to fractures (^∗^hip fracture 17%), often due to balance problems, bad eyesight, and slower reflexes. The list can be expanded but covers the most prevalent causes of death (for more details see Nilsson et al., 2002).

Among functional health indicators, studies show that grip strength is a reliable marker related to subsequent survival (e.g., Cooper et al., 2011; Syddall et al., 2017). Another test often used to characterize overall functional ability in older adults is the peak expiratory flow rate which is a brief measure of lung capacity (e.g., Proctor et al., 2006). Sensory functioning, including vision and hearing are often regarded as important markers of older adults’ capacity to maintain their functioning and coping with everyday life challenges, although eyeglasses and hearing aids nowadays frequently are used to overcome the age-related decline in vision and hearing. In our sample of 80 + year olds, the prevalence for hearing loss was as high as 61%, and as many as 49% were affected with cataracts, and 15% with glaucoma (see Nilsson et al., 2002). An interesting study by Wettstein et al. (2018), find support for an adaptive process of late-life sensory compensation, in which visual acuity can have a compensatory role to uphold cognitive ability especially when hearing impairment sets in.

Previous studies have shown that several psychological variables are associated with subsequent survival in older adults. In a review Johansson and Bjälkebring (2015), summarized hitherto research findings showing that cognitive abilities are highly associated with survival and longevity. Among the personality dimensions discerned in the five-factor model, conscientiousness is the dimension most strongly associated with longevity (e.g., Friedman et al., 2014). In addition, significant information about the likelihood for survival can be drawn from self-perceptions of aging and subjective life expectancy, which in turn provides evidence for individual’s ability to forecast their own longevity. Feelings of a purpose in life can increase survival (e.g., Hill and Turiano, 2014). Furthermore, positive dimensions of well-being are significantly associated with survival (e.g., Xu and Roberts, 2010). Life satisfaction and positive affect are not only well-being markers, but also potential predictors of longevity (Lyyra et al., 2006a).

Self-rated health is another important predictor and an indicator of a self-evaluation of overall health and vitality. Research findings underscore a significant association between self-rated health and longevity (e.g., Idler and Benyamini, 1997; Jylhä, 2009). Social connectedness and embeddedness are also positively related to longevity (Lyyra et al., 2006b), but the association seems stronger for closer and more emotionally rewarding social relationships, compared with contact frequencies (Holt-Lunstad et al., 2010). Johansson and Bjälkebring (2015), concluded that interaction patterns among these variables are likely and that various constellations can affect lifestyle and various health-related behaviors, which in turn affect the likelihood for maintenance of vitality, health, and thereby survival.

In the present study, we had two main aims. First, to provide an estimate of heritability of age of death from a population-based representative sample, conditioned on survival up to age 80. We derived such a heritability estimate to clarify to what degree genetic influences can account for individual differences in survival among individuals who already have survived into late life (i.e., more than 80 years). Given previous studies, demonstrating moderate heritability across the late half of the life span, and selection and age range of our sample, we expect to find relatively low heritability estimates. Our second aim was to evaluate the impact of various, and previously identified, predictors for survival among older adults, including those related to sociodemographic variables, genetics (i.e., APOE ε4), disease and health, lifestyle, cognitive health, functional markers, and personality characteristics. We conducted these analyses in an exploratory manner with the hope to clarify what matters, and what matters most, for subsequent survival among the oldest-old.

## Materials and Methods

### Sample

The OCTO-Twin Study (”Origins of Variance in the Old-Old”) (e.g., McClearn et al., 1997; Femia et al., 2001) was planned in the late 80’s and became the first major population-based longitudinal twin study directed to the oldest-old when the data collection started in 1991. Based on ubiquitous genetic influence demonstrated earlier in the lifespan, the study was originally designed to investigate whether the relative impact of genetic influences decrease, increase, or remain at the same magnitude for various aspects of health and biobehavioral functioning. Primary data was collected through in-person interviews and testing, supplemented by data from reviews of medical records. The longitudinal design encompassed five measurement occasions in which we included all twins alive who agreed to participate, irrespective of twin status (for details, see^2^).

The sample for the present analyses was originally drawn from the Swedish population-based Twin Registry. Dates of birth and date of death were obtained from official population registers. The OCTO-Twin data consisted of 149 identical/monozygotic and 202 same sex fraternal/dizygotic twin pairs. At baseline, the twins had to be born 1913 or earlier and both partners in the pair had to accept participation. That produced an overall pair-wise participation rate of 64% (for details see Simmons et al., 1997). At the first wave 351 twin pairs (149 MZ and 202 like-sex DZ pairs) were investigated (average age 83.6 years (SD = 3.2) with 67% women. In the current analyses, we excluded three individuals due to missing data on the data of death variable, leading to a sample size of 699.

### Measures and Procedure

The variables included in the assessment battery were originally selected for their demonstrated age-relevance, pertinence to different disciplinary perspectives in the gerontological literature and non-invasiveness. Measures were chosen to allow extension into advanced ages. The battery should be possible to administer while visiting participants in their own place of residency. A complete examination took at least 3–4 h, but a full day was reserved for each participant, which made it possible to arrange for coffee and lunch breaks. The assessments were performed by registered nurses (RNs) who visited the twins at their place of residence, whether in ordinary housing or in institutions.

The domains of interest included health and functional ability, mental health and cognition, personality, and interpersonal relationships. Blood samples were derived from twins with unknown zygosity and for various blood chemistry analyses and DNA extractions. We gathered supplement information by reviews of medical records. The broad set of variables selected for our present analyses and predictions of survival were categorized in the following manner:

*Sociodemographic information:* include sex, marital status (married, unmarried, widowed, divorced), education, using levels 1–8 reflecting stages of educational attainment (higher values indicate more years of formal schooling). The socioeconomic level was defined according to the social classification typically used and relevant for our cohort (taken three discrete values from 1 to 3, in which 1 represents upper class, and 3 working class). We approximated the childhood financial situation using the question: “When you grew up - how –well or badly- did money cover you family’s need?” (with the following response alternatives: 1 = badly, 2 = rather badly, 3 = rather well, and 4 = well). The adult life financial status was defined as a composite based on responses to the three questions: “How well did your money cover your and/or your family’s need? (1 = bad, 2 = rather bad, 3 = rather well, 4 = very well); “How well does your money cover your needs?” (1 = badly, 2 = rather badly, 3 = rather well, 4 = very well); “Is your present economic situation preventing you from doing what you like to do? (1 = Yes, to a great extent, 2 = Yes, to some extent, 3 = No).

*Genetics*: A study of twins allows comparison of resemblance in survival across monozygotic (MZ) and dizygotic (DZ) pairs, and thereby calculation of overall heritability. In addition, we determined the role of APOE ε4 from peripheral lymphocytes. The relative frequency of the e4/e4 homozygote was only 1.7% (*n* = 9), which made us to use a simplified classification of individuals as either e4 carriers (30.6%) or non-carriers in our analyses.

*Diseases and health related factors:*Nilsson et al. (2002) reported a review of medical records, self-reports, and registration of marker drugs for the analyzed sample to determine, verify, and uniformly classify more than 40 diseases into ICD-10 criteria (ICD, 1992). We included data on the following conditions in our present analyses: dementia, cancer of all types, thyroid disease, diabetes, B12 deficiency, cerebrovascular disease (CVD), osteoporosis, hip fracture, depression, Parkinson’s disease (PD), glaucoma, and cataract. For all these variables we coded them as 0 in the absence of such a diagnosis (over the lifespan) and 1 in the presence of a diagnosis. Systolic and diastolic blood pressure values were based on readings using a mercury sphygmomanometer. Body mass index was defined based on measured weight in kilograms divided by measured length in meters squared (i.e., BMI = kg/m^2^). Self-rated health was also included into this category, using a 4-item scale with the questions: “How do you evaluate your overall health condition?” (1 = good, 2 = about average, 3 = bad); “How do you rate your health compared to what it was 2-years ago?,” and “How do you rate your health compared to others of your own age?” (1 = better, 2 = about the same, 3 = worse). The fourth item was, “Do you think that your health-condition is preventing you from doing the things you would like to do?” (1 = not at all, 2 = partly, 3 = to a great extent). The internal consistency, as determined by Cronbach’s alpha, for this scale was 0.59.

*Lifestyle factors****:*** We included information on tobacco use over the life span (never, occasionally, former smoker, still smoker), and two questions with focus on previous and current intellectual engagement (“Do you/did you do anything in particular to “train your memory or keep your mind active”?” (0 = no, 1 = yes, to a certain degree, 2 = yes, definitely). Self-rated social embeddedness was defined based composite score derived from responses to four questions about quality and quantity of social contacts: “Have you got friends with whom you can talk?,” “Do you feel you are part of a set of friends?,” “Do you lack company?,” and “Do you feel abandoned?”. The response alternatives were, 0 = no, not at all, 1 = no, hardly, 2 = yes, to a certain degree, 3 = yes, to a high degree. The last two items were reversed before summing (alpha = 0.75).

*Cognitive health:* The first author (BJ) determined a global cognitive clinical rating, with scores ranging from 1–5. This rating was based on a thorough review of each participant’s baseline performance on a broad battery of memory and cognitive tests, relative to available norms, reference and cut-off values for each test, taking motor performance, sensory functioning, and compromised physical health into account. The review was supplemented with details and observations from the RNs’ who had administered the tests on site, which provided a comprehensive and clinically based categorization, rather than using a simplified metric approach for each measure. The categories were defined as: 1 = normal cognition; 2 = questionable cognitive impairment/MCI; 3 = mild cognitive impairment; 4 = moderate cognitive impairment; 5 = severe cognitive impairment. Categories 3–5 fully met criteria for dementia, according to DSM-III-R (American Psychiatric Association, 1987). The cognitive tests used to determine the score were: MMSE (Mini-Mental State Examination) measuring overall cognition, Information and Synonym used gauging crystallized abilities, Block Design, Figure Logic and Clock Test for fluid abilities, and Digit Symbol and a Perceptual Speed to test mental speed. Memory functioning was investigated by the Digit Span Forward and Backward test, and for episodic memory we administered a Prose Recall test, the MIR Memory Test, and the Thurstone’s Picture Memory test (see Hong et al., 2003). In addition, participants were asked to rate their own memory and cognitive functioning, using a summary scale based on the following, four questions: “Do you think - on the whole - that you have a good or a bad memory?” (1 = very good; 2 = good; 3 = rather good, 4 = neither good nor bad, 5 = rather bad, 6 = bad, 7 = very bad); “Do you think that you have any problems with your memory which make daily life more difficult?” (1 = no, not at all; 2 = no, hardly; 3 = hard to take a stand on; 4 = yes, to a certain degree; 5 = yes, definitely); “Do you think that your memory has changed during the last 2 years? (1 = improved; 2 = somewhat improved; 3 = neither better nor worse; 4 = somewhat impaired; 5 = impaired); “Do you think - on the whole - that you have good or bad cognitive ability (“presence of mind”)?” (1 = very good; 2 = good; 3 = rather good; 4 = neither good nor bad; 5 = rather bad; 6 = bad; 7 = very bad). Higher scores indicate poorer self-rated memory and cognition (alpha = 0.73).

*Functional markers:* In this predictor category, we included performance-based data from measures on grip strength and lung function. For grip-strength, we asked participants to squeeze a dynamometer six times, first three with the dominant and then three with the non-dominant hand. We used the mean value across these six trials as the grip strength score (alpha = 0.96). For lung function, we used data from a PEF-peak expiratory flow-measure in which participants are instructed to blow as much air as they can into a test tube. For more details on these tests see Proctor et al., 2006; Praetorius Björk et al., 2016; Duggan et al., 2019. We used the mean value across three PEF trials as lung function score (alpha = 0.94). For visual acuity and hearing, respectively, we use the self-reported categories of no problems, certain problems, severe problems, allowing for the use of compensatory aids (i.e., eyeglasses and hearing devices), and RNs’ evaluation of observed blindness/deafness.

*Personality characteristics and Life satisfaction:* We measured personality using the 19-item Eysenck Personality Inventory (EPI; Eysenck and Eysenck, 1964; see also Berg and Johansson, 2014), for the traits of neuroticism (alpha = 0.64) and extraversion (alpha = 0.74). The life satisfaction score was based on a 13-item version of the Life Satisfaction Index-A (Neugarten et al., 1961), with higher scores indicating higher satisfaction (for details, see Berg et al., 2011; alpha = 0.77). In addition, we also included a general measure of locus of control (with subscales for external (alpha = 0.59) and internal control (alpha = 0.62) and the Multidimensional Health Locus of Control (MHLC) scale specifically designed to measure health locus of control (with subscales for internal (alpha = 0.72), chance (alpha = 0.74), and powerful others (alpha = 0.75; for details see Rotter, 1966; Wallston and Wallston, 1982; Johansson et al., 2001). Higher values indicate more of the trait at focus.

Due to occasional missing data, the composite scores on the predictors in the above categories were weighted to have a score that reflects the proportion of provided data.

### Analysis

We defined the outcome in our analyses as the number of days alive, derived as the difference in days between day of birth and day of death. We then converted the days-alive variable to age of death in years, with six decimal places. There were no ties in the data. We then computed the heritability estimates using an ACE model (see e.g., Plomin et al., 2008), derived from full information maximum likelihood as implemented into the lavaan R package (Rosseel, 2012). Next, we evaluated the conditional proportional mortality-related hazard rates using Cox regression (survival) models as implemented in the survival R package (Therneau, 2021). In all survival models, we accounted for late entry to the risk set by modeling the proportional hazard rate simultaneously as a conditional base function of both age of death and age at baseline. We accounted for dependency as relating to the twin design by a Gaussian frailty component (i.e., random effects).

In all fitted survival models, we included age at baseline, sex, and education as predictors. Then, in a first step, we added covariates, or factors, separately as predictors. This, in order to estimate their contributions to the hazard rate independently (i.e., beyond the effects of age at baseline, sex, and education). Then, in a second step, we combined similar type of categories of predictors (i.e., sociodemographic, diseases and health related factors, life-style, cognitive health, functional markers, and personality and life-satisfaction characteristics) into the same model in order to compare their conditioned contribution to the hazard rate. As a third step, we combined the strongest predictors (based on *p*-value at least less than 0.05) from each category into the same model to evaluate their conditioned contribution across the categories. If two variables overlapping in content (e.g., cognitive status and dementia diagnosis) and reached the cut-off value, we included the variable with the largest overall effect size. To test the proportional assumption in the survival models we plotted the Schoenfeld residuals (Schoenfeld, 1982) as a function of ranked age of death and superimposed a smooth summary function as well as computed the correlation between the residuals and ranked age of death.

## Results

**FIGURE 1:**
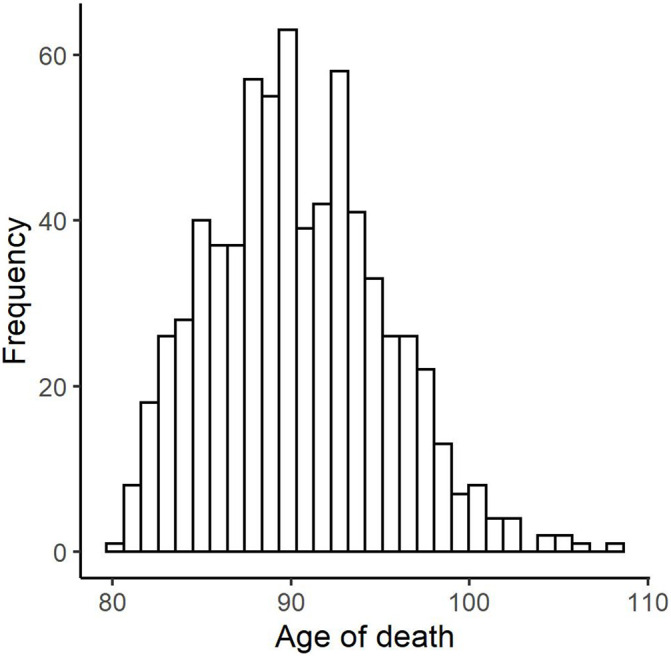
Histogram of age of death in the OCTO-Twin study.

Descriptive for all variables included in the analyses are shown in Table 1. In Figure 1, we plot the distribution of the age of death variable for the total sample (*N* = 699, M = 90.39, SD = 4.84, minimum = 80.24, maximum = 108.25; skewness = 0.37; kurtosis = −0.06).

**TABLE 1:** Descriptive for the OCTO-Twin sample used in the analyses.

**Variables**	***N***	**M (*SD;min/max^a^*) or n (%)**
**Sociodemographic indicators**		
Age of death	699	90.39 (4.84; 80.24/108.25)
Sex (females)	699	466 (66.67)
Age at T1	699	83.60 (3.17; 79.27/97.92)
Zygosity (MZ)	699	296(42.35)
Marital status	684	
Married		211(30.85)
Unmarried		92(13.45)
Widowed		356(52.05)
Divorced		25(0.037)
SES	659	
Group 1		85(12.75)
Group 2		254(38.54)
Group 3		321(48.71)
Economy	649	3.00(0.45; 1.00/4.00)
Childhood economy	647	
Bad		86(13.29)
Rather bad		111(17.16)
Rather good		254(39.26)
Good		196(30.29)
**Genetics**		
APOE e4	538	165(30.67)
**Diseases and health indicators**		
Self-rated health	664	1.80 (0.50; 1.00/3.00)
Dementia	699	225 (32.19)
Cancer	699	181(25.89)
Thyroid disease	699	106(15.16)
Diabetes	699	74(10.44)
B12 deficiency	699	121(17.31)
Cerebrovascular lesions (CVD)	699	165(23.60)
Osteoporosis	699	78(11.15)
Hip fracture	699	121(17.31)
Parkinson’s disease	699	25(4.58)
Depression	699	140(20.03)
Glaucoma	699	80(11.44)
Cataracts	699	337(48.21)
Blood pressure systolic	627	159.23(22.86; 100.00/260.00)
Blood pressure diastolic	629	83.27(11.89; 50.00/160.00)
Body mass index	575	24.47(3.81; 12.62/38.57)
**Life-style**		
Smoking	661	
No, never tried		403(60.97)
Yes, now and then (e.g., socially)		43(6.51)
Yes, but has quit		161(24.36)
Yes, still smokes		54(8.17)
Social embeddedness	613	9.13(2.62; 0.00/12.00)
Intellectual engagement	644	0.84 (1.24; 0.00/4.00)
**Cognitive health**		
Self-rated memory	663	2.59 (0.83; 1.00/7.00)
Cognitive status	695	1.76 (1.13; 1.00/5.00)
**Functional markers**		
Grip strength	579	0.55 (0.19; 0.05/1.22)
Lung function (PEF)	442	286.52(105.19; 66.67/600)
Vision	679	
Blind		11(1.62)
Severe problems		63(9.28)
Certain problems		143(21.06)
No problems		462(68.04)
Hearing	685	
Deaf		1(0.20)
Severe problems		62(9.05)
Certain problems		236(34.45)
No problems		386(56.35)
Personality characteristics		
Extraversion	487	0.56(0.24; 0.00/1.00)
Neuroticism	486	0.29(0.25; 0.00/1.00)
Life-satisfaction	479	3.62(0.64; 1.62/5.00)
Locus of control external	468	3.57(0.68; 1.50/5.00)
Locus of control internal	470	3.72(0.67; 1.75/5.00)
Health locus of control internal	472	3.43(0.81; 1.00/5.00)
Health locus of control chance	470	3.32(0.91; 1.00/5.00)
Health locus of control powerful others	471	3.72(0.85; 1.00/5.00)

*^a^Minimum and maximum value.*

### Heritability of age of Death After age 80

**FIGURE 2:**
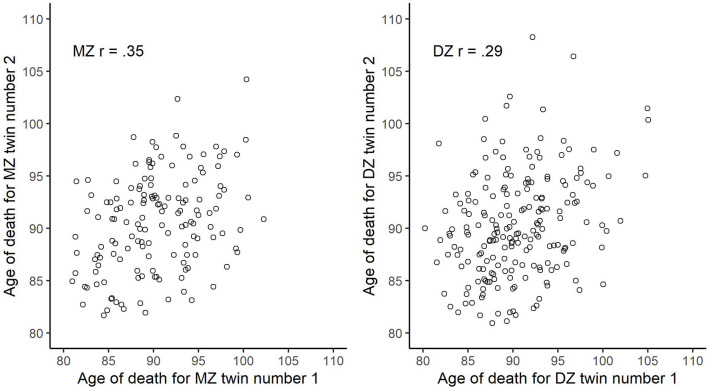
Bivariate scatter plot of age of death across randomly drawn within-twin-pair individuals stratified by zygosity.

In Figure 2, we plot the bivariate distribution of age of death across randomly drawn within-twin-pair individuals as stratified by zygosity. The bivariate correlation was 0.35 for the MZ and 0.29 for the DZ. Using Falconer’s equation, we derived at the broad-sense heritability of 12% {i.e., 100×[2×(0.35–0.29)]}. We present descriptive and variance/covariance matrices for age of death, as stratified by zygosity, in Table 2. Next, fitting an ACE model to the data, using maximum likelihood estimator, provided the following estimates: A^0.5^ = 0.341, 95% CI [−0.03, 0.72]; C^0.5^ = 0.48, 95% CI [0.27, 0.69]; E = 0.65, 95% CI [0.51, 0.75]. This implies (in agreement with the Falconer’s estimate) that about 12% of the between-person variability in age of death, conditioned on being alive at age 80, can be ascribed to genetic influences, while about 23% to common/shared environment, and about 65% to unique environmental exposures.

**TABLE 2:** Descriptive and variance/covariance matrices for age of death as stratified by zygosity.

**MZ (M = 90.41, SD = 4.77)**		**DZ (M = 90.38, SD = 4.91)**	
24.45		23.42	
7.90	21.07	6.99	24.82

*Variance on diagonals and covariance on off-diagonals.*

### Survival as a Function of Gender, age at Baseline, and Education

**FIGURE 3:**
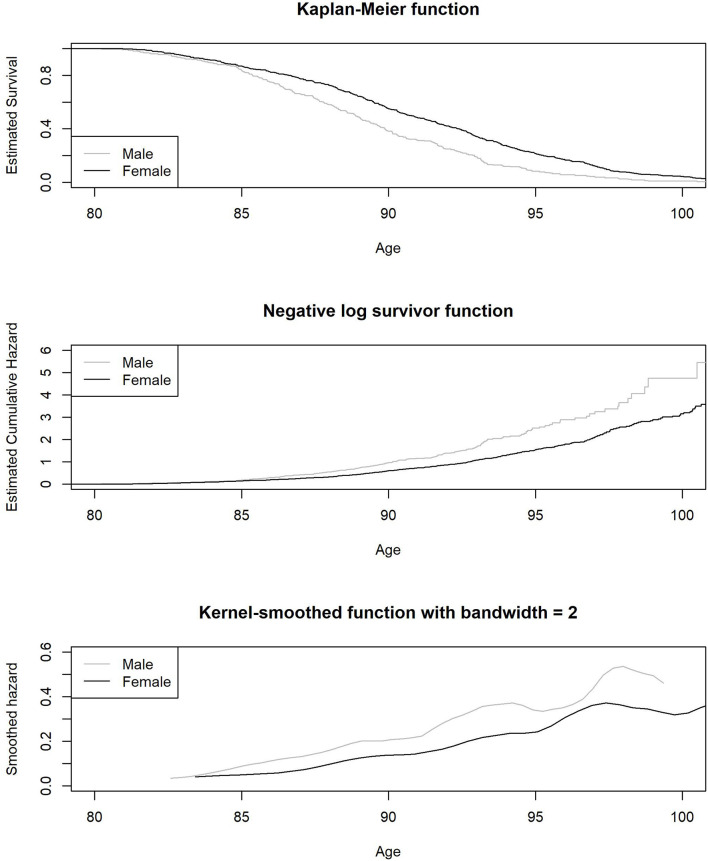
Estimated Kaplan-Meier survival function, negative logarithm of the survival.

The average age of death for males was 89.10 (SD = 4.17) and for females 91.04 (SD = 5.03). In Figure 3, we plot the Kaplan-Meier estimated survival function, negative logarithm of the survival function, and the Kernel smoothed hazard rate function for age at death as stratified by sex. As expected, the hazard rate was consistently higher for males. The proportional hazard function estimates, as derived from the Cox regression model, are shown under Model 1 in Table 3, while controlling for age at baseline and education. The mortality related hazard rate was higher among males by a factor of 1.59 (i.e., 1/0.63). Each additional year for age at baseline was associated with a reduction in the hazard rate by a factor of 0.95, implying baseline selection (we note that we account for late entry to the risk set as part of the baseline hazard function). Education was unrelated to the hazard rate.

**TABLE 3:** Parameter estimates from Cox proportional hazard models with age of death as outcome.

**Model**	**Parameter**	**Est.**	**SE**	**exp(est.)**	**95% CI**
1	Age at T1	−0.055***	0.015	0.95	[0.92, 0.98]
	Sex	−0.464***	0.097	0.63	[0.52, 0.76]
	Education	–0.030	0.028	0.97	[0.91, 1.03]
2^a^	Marital status (married as ref.)				
	Unmarried	0.111	0.132	1.12	[0.84, 1.49]
	Widowed	0.079	0.107	1.08	[0.88, 1.33]
	Divorced	0.749**	0.243	2.09	[1.30, 3.37]
3	SES (Group 1 as ref.)				
	Group 2	–0.047	0.145	0.95	[0.71, 1.27]
	Group 3	0.089	0.151	1.09	[0.81, 1.47]
4	Economy	–0.071	0.106	0.93	[0.76, 1.15]
5	Childhood economy	0.049	0.048	1.05	[0.96, 1.15]
6	APOE e4	0.239*	0.118	1.27	[1.01, 1.60]
7	Self-rated health	0.600***	0.091	1.82	[1.53, 2.18]
8	Dementia	0.454***	0.094	1.58	[1.31, 1.89]
9	Cancer	0.150	0.098	1.16	[0.95, 1.41]
10	Thyroid disease	−0.281**	0.123	0.76	[0.59, 0.96]
11	Diabetes	0.333**	0.142	1.39	[1.06, 1.84]
12	B12 deficiency	–0.110	0.111	0.90	[0.72, 1.11]
13	CVD	0.808***	0.102	2.24	[1.84, 2.74]
14	Osteoporosis	–0.032	0.140	0.97	[0.74, 1.28]
15	Hip fracture	0.419***	0.116	1.52	[1.21, 1.91]
16	Parkinson’s disease	0.527*	0.225	1.69	[1.09, 2.63]
17	Depression	0.013	0.106	1.01	[0.82, 1.25]
18	Glaucoma	–0.168	0.133	0.845	[0.65, 1.10]
19	Cataracts	−0.208*	0.088	0.81	[0.68, 0.96]
20	BP^b^ systolic	–0.041	0.025	0.96	[0.91, 1.01]
	BP diastolic	0.108*	0.046	1.11	[1.02, 1.22]
21	Body mass index	−0.030*	0.014	0.97	[0.95, 0.99]
22	Smoking	0.117*	0.049	1.12	[1.02, 1.24]
	Social embeddedness	−0.066***	0.018	0.94	[0.90, 0.97]
23	Intellectual engagement	−0.095*	0.037	0.91	[0.84, 0.98]
24	Self-rated memory	0.262***	0.057	1.30	[1.16, 1.45]
25	Cognitive status	0.353***	0.042	1.42	[1.31, 1.55]
26	Grip strength^c^	−0.148***	0.032	0.86	[0.81, 0.91]
27	Lung function^d^	−0.127*	0.062	0.88	[0.78, 0.99]
28	Vision	−0.160**	0.061	0.85	[0.76, 0.96]
29	Hearing	–0.034	0.072	0.97	[0.84, 1.11]
30	Extraversion	0.067	0.225	1.07	[0.69, 1.66]
	Neuroticism	0.194	0.225	1.21	[0.78, 1.89]
31	Life-satisfaction	−0.372***	0.086	0.69	[0.58, 0.82]
32	Locus of control external	0.119	0.088	1.13	[0.95, 1.34]
	Locus of control internal	–0.044	0.088	0.96	[0.81, 1.14]
33	Health LoC internal	0.006	0.075	1.01	[0.87, 1.17]
	Health LoC chance	0.011	0.067	1.01	[0.89, 1.15]
	Health LoC powerful others	0.051	0.073	1.05	[0.91, 1.22]

*^*a*^In all comparisons below we accounted for age at baseline, sex, and education.*

*^*b*^BP was scaled by a factor of 10.*

*^*c*^Grip strength was scaled by a factor of 10.*

*^*d*^Lung function was scaled by a factor of 100.*

*p < 0.05; **p < 0.01; ***p < 0.001.

### What Matters? the Role of Various Predictors for Subsequent Survival

In subsequent models, presented as part of Table 3, we accounted for sex, age at baseline, and education, while separately adding selected covariates or factors to the model. This in order to evaluate the specific contributions to the mortality hazard function. We included marital status as four level factor using “married” as reference category. Being divorced was associated with an elevated hazard function by a factor of 2.09 in comparison to the married group. The hazard rate for the unmarried and widowed group was substantially smaller. We note, however, that there were only 25 individuals in the divorced group. Socio-economic status (SES), economy, and childhood economy were unassociated with the hazard rate.

As expected, the APOE e4 allele was associated with an elevated hazard rate by a factor of 1.27. Poorer self-rated health was also associated with an increased hazard rate. Additional standard deviation on this scale raised the hazard by a factor of 1.35. As expected, both dementia diagnosis and cerebrovascular disease elevated the mortality hazard, by a factor of 1.58 and 2.24, respectively. Diabetes was associated with higher rate by a factor of 1.39 and Parkinson’s disease by a factor of 1.69. Neither the cancer category, B12 deficiency, osteoporosis, nor glaucoma were associated with mortality in this model. Hip fracture was, however, associated with an elevated risk by a factor of 1.52. Somewhat surprisingly, both a diagnosis of thyroid disease and cataracts were associated with lower mortality risk, by factors of 0.76 and 0.85, respectively.

We included both diastolic and systolic blood pressure simultaneously into the model. The diastolic, but not systolic, measure was associated with mortality, such that, additional standard deviation on the diastolic measure increased the hazard rate by a factor of 1.14. The BMI was negatively associated with the hazard risk, such that, additional standard deviation reduced the rate by a factor of 0.89. Further, additional value on the smoking scale was associated with an increase in the hazard rate by a factor of 1.12. Participation in intellectual engagement and social embeddedness were also related to a reduced mortality hazard; a standard deviation on these scales reduced the hazard by a factor of 0.89 and 0.84, respectively. Both poorer self-rated memory and cognitive status were associated with a substantial increment in the mortality hazard rate. An additional standard deviation on these scales produced a higher hazard rate by a factor of 1.24 and 1.49, respectively.

Grip strength, lung function, and vision (but not hearing), showed a negative association with the hazard rate, implying that an additional standard deviation on these variables reduced the hazard by a factor of 0.89, 0.85, and 0.89, respectively. None of the personality measures were associated with the mortality hazard except for life-satisfaction which had a negative association; an extra standard deviation on this scale reduced the hazard function by a factor of 0.79.

### What Matter Most? Comparing the Role of Various Predictors for Subsequent Survival Within and Across Categories

We expanded our analyses by including similar types of predictors into the same model. First, we combined (in addition to sex, age at baseline, and education) the sociodemographic variables. That is, marital status, SES, economy, and childhood economy to one model. The SES and economy related variables still showed little or no association with the hazard risk. But, being divorced was still (β = 0.70, *SE* = 0.24; exp(β) = 2.02, 95% CI [1.19, 3.42]) associated with an elevated hazard risk, despite controlling for these other predictors.

In a second step, we combined the various disease and health-related indicators into the same model. The estimates from this model are shown in Table 4. Noteworthy, despite control for health-related variables, self-rated health remained as a strong predictor of the mortality risk, with only a drop in the effect size from 1.69 to 1.39 (i.e., from model in Table 3). The effect size for thyroid disease remained approximately similar, although below the significant level. This was due to less precision in the estimation of this parameter, given a smaller sample size and missing data on the other covariates. The effect sizes for diabetes largely remained, although reduced from 1.39 to 1.25. The hip fracture, effect size showed a reduction from 1.52 to 1.27. The effect size for Parkinson’s was reduced from 1.69 to 1.51. The effect sizes for cataract, however, remained relatively unaffected, as well as for BMI.

**TABLE 4:** Parameter estimates from Cox proportional hazard models with age of death as outcome and health-related variables as predictors.

**Parameter**	**Est.**	**SE**	**exp(est.)**	**95% CI**
Age at T1	−0.129***	0.021	0.88	[0.84, 0.91]
Sex	−0.441***	0.127	0.64	[0.50, 0.83]
Education	–0.009	0.034	0.90	[0.93, 1.06]
Self-rated health	0.527***	0.114	1.69	[1.35, 2.11]
Dementia	0.387***	0.117	1.47	[1.17, 1.85]
Cancer	0.32**	0.118	1.38	[1,10, 1.74]
Thyroid disease	–0.268	0.143	0.77	[0.58, 1.01]
Diabetes	0.223	0.174	1.25	[0.89, 1.76]
B12 deficiency	–0.143	0.134	0.87	[0.67, 1.13]
CVD	0.631***	0.132	1.88	[1.45, 2.44]
Osteoporosis	–0.089	0.172	0.92	[0.65, 1.28]
Hip fracture	0.236	0.156	1.27	[0.93, 1.72]
Parkinson’s disease (PD)	0.410	0.280	1.51	[0.87, 2.62]
Depression	–0.056	0.130	0.95	[0.73, 1.22]
Glaucoma	0.019	0.155	1.02	[0.75, 1.38]
Cataracts	–0.213	0.110	0.81	[0.65, 1.00]
BP^a^ systolic	–0.033	0.028	0.97	[0.92, 1.02]
BP diastolic	0.131**	0.051	1.14	[1.03, 1.26]
Body mass index	−0.045*	0.015	0.96	[0.93, 0.98]

*^*a*^BP is scaled by a factor of 10.*

*p < 0.05; **p < 0.01; ***p < 0.001.

In a third step, we included the life-style predictors (i.e., smoking, social embeddedness, and intellectual engagement) simultaneously into the same model. This model revealed that the effect sizes of smoking (β = 0.112, *SE* = 0.052; exp(β) = 1.12, 95% CI [1.01, 1.24]) and social embeddedness (β = −0.06, *SE* = 0.019; exp(β) = 0.94, 95% CI [0.90, 0.97]) remained largely unaffected, compared with the coefficient estimates reported in Table 3. The effect size for intellectual engagement (β = −0.068, *SE* = 0.039; exp(β) = 0.93, 95% CI [0.85, 1.01]) was, however, reduced to a minor extent. In the next model, we simultaneously added subjective memory and cognitive status to the same model. This model revealed minor differences in estimates for subjective memory (β = 0.121, *SE* = 0.062; exp(β) = 1.13, 95% CI [1.00, 1.27]), but not for cognitive status (β = 0.364, *SE* = 0.057; exp(β) = 1.44, 95% CI [1.28, 1.61]).

In the fourth step, we implemented a model including all functional markers simultaneously. Estimates from this model revealed a substantial drop in effect sizes for grip strength (β = −0.068, *SE* = 0.039; exp(β) = 0.94, 95% CI [0.87, 1.01]), lung function (β = −0.099, *SE* = 0.064; exp(β) = 0.91, 95% CI [0.80, 1.03]), and vision (β = 0.133, *SE* = 0.090; exp(β) = 0.88, 95% CI [0.73, 1.05]). The effect size for the hearing variable was still minor and non-significant.

In a fifth step, we included all personality related predictors into the same model. Estimates from this model revealed that life satisfaction remained as the only significant predictor (β = −0.526, *SE* = 0.104; exp(β) = 0.59, 95% CI [0.48, 0.72]), while the others were relatively unaffected and still non-significant.

In the final step, addressing the question what matters most, we simultaneously included selected predictors from all the different categories into the same model. The estimates from this model are shown in Table 5. Self-rated health, cancer, cerebrovascular disease, diastolic BP, cognitive status, and life-satisfaction all remained as significant predictors of the mortality-related hazard (i.e., in addition to sex and age at baseline). The effect sizes of BMI, smoking, social embeddedness, and vision were, however, reduced substantially.

**TABLE 5:** Parameter estimates from Cox proportional hazard models with age of death as outcome and a combination of variables from different categories as predictors.

**Parameter**	**Est.**	**SE**	**exp(est.)**	**95% CI**
Age at T1	−0.128***	0.024	0.88	[0.84, 0.92]
Sex	−0.497***	0.146	0.61	[0.46, 0.81]
Education	–0.003	0.036	1.00	[0.93, 1.08]
Self-rated health	0.332**	0.136	1.39	[1.07, 1.82]
Cancer	0.286*	0.126	1.33	[1.04, 1.70]
CVD	0.722***	0.151	2.06	[1.53, 2.77]
Diastolic BP^a^	0.100*	0.048	1.11	[1.01, 1.22]
BMI	0.103	0.064	0.98	[0.95, 1.01]
Smoking	0.103	0.064	1.11	[0.98, 1.26]
Social embeddedness	–0.003	0.027	0.99	[0.95, 1.06]
Cognitive status	0.292**	0.747	1.34	[1.07, 1.68]
Vision	0.004	0.097	1.00	[0.83, 1.22]
Life-satisfaction	−0.225*	0.103	0.80	[0.65, 0.98]

*^*a*^Diastolic BP is scaled by a factor of 10.*

*p < 0.05; **p < 0.01; ***p < 0.001.

## Discussion

In this study, we addressed the questions of what matters and what matters most for survival after age 80. We based our analyses on data from a population-based twin sample of monozygotic (identical) and same-sex fraternal (dizygotic) twins followed from age 80, until death. The fact that we conducted our analyses using a select sample of hardy survivors, born more than 100 years ago, should be considered when comparing our findings of predictions and for their relevance at younger ages. The observed median life expectancy (age at which 50% of a birth cohort is alive) for those born in Sweden during the period 1893–1913 was in the range of 65–72 years for males and for 70–79 years for females. The expectancy for the individuals in our birth cohort to be alive at age 80 and beyond was only in between 2.5–6% for males and 8.5–9.2% for females (see SCB, 2020). This remark, concerning generation and cohort differences, is important to consider in efforts to identify and determine the relative impact of various mortality-related predictors. In this respect, we may find that longevity predictors can vary in type or differ in magnitude considerably across birth cohorts, which needs to be considered when comparing findings from a sample born more than 100 years ago with data from more recent birth cohorts. Furthermore, predictors of longevity, which are informative and relevant from an early age, are not necessarily valid to predict subsequent survival for those who have survived into a later stage of life. This was evident in our study by the fact that SES and financial status no longer acted as predictors for survival, as would be expected in younger samples.

### The Role of Sociodemographic for Survival

Studies typically find that SES and education act as relatively strong predictors for longevity (e.g., Stringhini et al., 2017; Steptoe and Zaninotto, 2020). However, we could not replicate these findings, which likely reflect a restricted education range in our sample as well as greater homogeneity in overall socioeconomic status. Later born cohorts of late life survivors may therefore show other associations with these two common survival markers. Age at baseline was positively associated with subsequent survival. This infers that, given comparison of the hazard rate at a specific age (e.g., age 91) those that accepted study participation at later ages showed a lower expected hazard rate. This finding inform us that those who entered the study at a higher age in fact represent “the even more hardy ones” who will survive even longer than their counterparts who accepted participation at younger ages. Less surprising was our finding that women tend to live longer. For marital status, we only found that our small sample of divorced individuals showed a higher mortality risk. However, this finding needs to be replicated in samples with a higher frequency of divorced individuals, although our finding is in line with previous reports on the lethal consequences of divorce (e.g., Norgård Berntsen and Kravdal, 2012).

### The Role of Genetics for Survival

The analysis revealed a heritability estimate of about 12%, which is a lower estimate than previously reported in older adults (e.g., Christensen et al., 2006). This corresponds to claims that the heritability for subsequent survival is likely to be higher in the younger age range. However, Ruby et al. (2018) reported that the heritability for birth cohorts across the 1800s and early 1900s is rather well below 10%. As expected, we could confirm the significant role of APOE status. Thus, the association with the APOE e4 allele remained in late life, as those with a e4 allele had a shorter remaining life span, compared with non e4 carriers (e.g., Wolters et al., 2019). Notably, in complementary analyses (not reported), the APOE effect was reduced (β = 0.048, *SE* = 0.122; exp(β) = 1.05, 95% CI [0.83, 1.33]) to non-significance when we accounted for cognitive status.

### The Role of Diseases and Health Related Factors for Survival

Among the many analyzed diseases, we confirm strong expected associations for dementia, cerebrovascular disease, diabetes, Parkinson’s disease, and history of hip fracture. The effect sizes for dementia, CVD, diastolic BP, and BMI remained relatively unaffected when we controlled for comorbidities. The hip fracture effect replicates previous findings of an excess mortality risk after a hip fracture that last over many years (e.g., von Friesendorff et al., 2016). This frailty may be associated with immobility preventing a physically active and healthier lifestyle. The effects sizes for hip fracture, as well as for diabetes and Parkinson’s disease, were substantially reduced when we controlled for comorbidity (see Table 4).

More surprisingly, we found that the presence of thyroid disease predicted longer survival in our sample, which awaits further investigations, as both subclinical hypothyroidism and hyperthyroidism previously have been associated with an increased mortality risk (e.g., Ochs et al., 2008). A similar positive survival effect was found for cataract. These paradoxical findings may be explained as selection effects. We can speculate whether individuals receiving diagnosis for these conditions are more vital and more demanding for an appropriate treatment. Interestingly, the predictive value of both thyroid disease and cataract remained relatively unaffected even after controlling for all other diseases (see Table 4), which means that these unexpected results are not accounted for by comorbidities. Also, given that we accounted for cognitive status, the thyroid disease effect size remained similar (β = −0.250, *SE* = 0.126; exp(β) = 0.78, 95% CI [0.61, 0.99]). The effect size for cataract, however, was reduced somewhat (β = −0.092, *SE* = 0.091; exp(β) = 0.91, 95% CI [0.76, 1.09]).

Depression was not related to subsequent survival, which was an unexpected finding given that many studies show that depression substantially increases the mortality risk (e.g., Wulsin et al., 1999), and that late-life depression is associated with higher risk of both all-cause and cardiovascular mortality (Wei et al., 2019). A possible explanation for our finding is that our depression diagnosis is likely to reflect compromised mental health at earlier ages, rather than in later life.

Further, we found that higher diastolic blood pressure, but not systolic, was associated with a shorter survival. This is in line with previous studies showing that higher systolic blood pressure in older ages can be compensatory and in fact associated with better survival, while diastolic pressure is negatively related to all-cause mortality (e.g., Satish et al., 2001). We also found that higher BMI in fact was protective and associated with longer survival. Notably, few individuals were overweight in our sample. Our finding corresponds to previous reports of a U-shaped association between BMI and all-cause mortality (e.g., Cheng et al., 2016). In fact, when we modeled the hazard rate as a conditional function of an additional quadratic BMI component, we received the following estimate, β = 0.005, *SE* = 0.002; exp(β) = 1.005, 95% CI [1.003, 1.010], and a linear component, β = −0.296, *SE* = 0.128; exp(β) = 0.74, 95% CI [0.58, 0.96], implying a non-linear U-shaped association. A low BMI is typically found to be accompanied with an increased mortality risk which in our sample indicate compromised overall health.

Notably, cancer was not a significant predictor when we only controlled for baseline age, sex and education (shown in Table 3, with an effect size of 1.16). However, when we controlled for other health-related variables and diseases, the effect size became substantially larger, i.e., 1.38 and 1.33, respectively (see Tables 4, 5). This finding implies a suppression effect, which may reflect the broad malignancy category with several cancer types among our cancer survivors (26%), offered life-promoting treatments. Another explanation relates to comorbidities (e.g., dementia, CVD) that initially hid the effect of cancer.

Our findings largely correspond to previous studies demonstrating differential survival related to various disease conditions in later life. The results also confirm numerous studies showing that self-rated health is an informative marker for subsequent survival. Those who evaluate and self-diagnose their health as better also tend to live longer (e.g., Lyyra et al., 2006a; Feenstra et al., 2020). We may perhaps find it remarkably that self-rated health remains a relatively strong predictor of mortality (e.g., Jylhä, 2009), even when we control for multiple health related variables (seen in a comparison of effect sizes in Tables 3, 4 where the effect size only dropped from 1,82 to 1.69). The association between self-rated health and mortality cannot be fully accounted for by individual differences in cognitive status or personality-related variable like life-satisfaction (as shown in Table 5, were the effect size dropped to 1.39). As previously emphasized, self-rated health reflects a broader assessment of own health and functioning with reference to age-fellows, rather than experiences of a disease burden (Strawbridge and Wallhagen, 1999).

### The Role of Lifestyle Factors for Survival

Smoking was, as expected related to shorter survival. More interestingly, we found that self-reported intellectual engagement and social embeddedness also predicted subsequent survival, pointing toward the importance of maintaining social life and acquiring as well as preserving knowledge for making life worth living. An interesting study in this context, focusing on the valuation of life and more specifically on active attachment showed that old and very old individuals differ in terms of endorsement and with respect to what makes a life worth living. Whereas health factors were more important among the young-old, social factors were more important in the old-old group (Jopp et al., 2008). Our findings support and extend this interpretation in the context of survival.

### The Role of Cognitive Health for Survival

Our cognitive status indicator revealed a clear pattern showing that those with better cognition also tended to live longer, which partly was accounted for by the fact that individuals categorized as 3–5 met the dementia criterion. Noteworthy, better self-rated memory was also positively associated with survival. It is by now repeatedly shown that cognitive impairment and decline is indicative for a shorter life span, specifically demonstrated in terminal cognitive decline trajectories for various cognitive abilities (e.g., Thorvaldsson et al., 2008).

### The Role of Functional Markers for Survival

Among the functional markers, we found that the measures of grip strength and lung function were associated with subsequent survival; those with better performance on these two measures lived longer. This confirm previous findings, for example, McGrath et al. (2018), who showed that decreased handgrip strength was associated with ADL limitations and higher hazard for mortality. Our finding that better self-evaluated visual acuity was positively associated with survival is also in line with studies showing that worse visual acuity is indicative of a higher mortality rate (e.g., Freeman et al., 2005). Hearing was not a significant marker for mortality in our study, which may reflect that relatively few individuals were afflicted with serious hearing loss, preventing everyday coping and interactions in social life. Notably, when we included all the functional markers into the same model the effect size dropped for all variables. This may reflect that similar underlying neurophysiological mechanism can be responsible for the mortality-related associations across these markers, which is in line with the common cause assumption (e.g., Christensen et al., 2001) of aging degeneration.

### The Role of Personality Characteristics and Life Satisfaction for Survival

Among the examined markers in this category of potential predictors, we only found that life-satisfaction to be positively associated with a longer subsequent survival. This result is in line with several studies (e.g., Sadler et al., 2012; Hülür et al., 2017). However, compared with findings reported by Hülür et al. (2017), we found no associations with our measures of personal control (general or health related locus of control) and survival, which partly may reflect that those scales were only taken by a select portion of individuals, able to comprehend and return the inventories.

### Multiple Predictors in Concert and Survival

A strength in the present study is that it allowed a simultaneous examination of the potential role among multiple predictors. Following the first step of identifying potential predictors, “what matters,” we then turned to the question of “what matters most”? In doing so, it is important to remember that human functioning is highly inter-related, which make it unlikely to find isolated health conditions and other markers associated with late life survival. Interestingly, we could anyhow identify that some diseases categories, for example cerebrovascular disease and dementia, remained strong predictors in preventing a more extended life span after age 80. In the same manner, we found that self-rated health to be a strong survival indicator and that life satisfaction acted as positive marker for subsequent survival in advanced ages.

Although it would seem attractive to present a ranking list in response to the question of “what matters most,” it is also important to realize that many of the candidate variables evaluated in this study were inter-correlated. Therefore, the specific effect sizes were often substantially affected by a simultaneous inclusion of several variables into the same model. In addition, scale characteristics and metric properties (such as reliability and validity), differ across measures, rendering the comparison even more difficult. We therefore hesitate to provide a detailed weight for what matters most. However, as seen in Table 5, our analyses provide strong support for a shortlist that encompasses cerebrovascular disease, cognitive status, self-rated health, and life-satisfaction, in addition to the expected survival advantage among women, non-smokers, and non-carriers of the APOE-e4 allele. Our finding of an overall heritability estimate of 12% also emphasize the importance of multiple non-genetic influences for late life survival.

### Strengths and Limitations

Certain limitations and strengths merit comments. First, our sample was comprised of late life twin survivors born in the late 1800 and at the beginning of the 19th century. To test for potential selection effects due to twin ship, we compared our twin sample with a population-based community sample of non-twins largely in the same age range for health and overall functioning (Simmons et al., 1997). In this analysis, one member of each twin dyad was randomly selected. Adjustments for age, sex, and type of housing reveled significant differences only in three out of 20 comparisons, in which the twins were more advantaged in health and bio-behavioral functioning. The conclusion from this comparison was that twin pairs surviving into very late life are largely similar to a representative sample of non-twins of the same age (Simmons et al., 1997). Furthermore, the unique experiences and exposures in our select cohort born more than hundred years ago are unlikely to be similar to that of later cohorts in which the likelihood for survival have increased considerable over the years. Despite this important remark, the predictors identified in our sample are likely to be valid also for later born individuals, although this claim needs clarification in empirical studies. Second, the validity and reliability of our predictors varied, with some relatively brief indices (e.g., a medical history of having or not meeting a certain diagnostic category, without severity accounted for) while others reflected more detailed measurements (e.g., grip strength, lung function, blood pressure, and BMI). Third, our predictors do not cover all potential markers, although we originally selected them based on gerontological relevance for a broad population-based longitudinal study. Fourth, we did not examine additive or multiplicative effects of having multiple diseases (i.e., multimorbidity) which was beyond the scope of the present study.

Despite these potential shortcomings, the strength of our study refers to the fact that we were able to use a rich and comprehensive data set gathered in a population-based sample of twins examined in–person for a whole day over a broad range of variables. This allowed analyses of the overall research question of what matters for subsequent survival past age 80 as well as analysis of heritability. Of special importance is the fact that our study encompasses detailed and valid information drawn from official register data on exact date of birth, as well as date of death.

## Conclusion

In our study, we addressed the questions of what matters and what matters most for survival in late life. Besides a survival advantage of being a female, our findings point to the importance of staying unaffected by certain diseases, for example cerebrovascular disease and compromised cognition. In this respect, we need to focus on how to extend the health-span in which older adults can enjoy their life free of devastating disease and thereby disabilities and compromised overall functioning. Furthermore, we confirmed that the deceptively simple marker of self-rated health is a strong predictor for subsequent survival and that it measures much more than only disease burden. We also found that life satisfaction in late life is an informative marker for subsequent survival. In that respect, we need to ensure that late life is accompanied not only with years of survival, but also by quality of life.

Our study largely confirms the early findings of Palmore (1969) and stresses his recommendations for how to increase longevity by maintaining a useful and satisfying role in society, preserving good physical functioning, not smoking, and conserving a positive view of life. Finally, what often may be considered as “soft markers” for health, functioning, and survival provided in fact substantial information about the likelihood for surviving beyond an already advanced age. Notably, these markers also reflect to what degree older adults find life worth living.

## Data Availability Statement

The original contributions presented in the study are included in the article/supplementary material, further inquiries can be directed to the corresponding author/s.

## Ethics Statement

The studies involving human participants were reviewed and approved by an Ethical Board at the Karolinska Institute, Sweden. Written informed consent for participation was not required for this study in accordance with the national legislation and the institutional requirements.

## Author Contributions

Both authors listed have made a substantial, direct and intellectual contribution to the work, and approved it for publication.

## Conflict of Interest

The authors declare that the research was conducted in the absence of any commercial or financial relationships that could be construed as a potential conflict of interest.

## Publisher’s Note

All claims expressed in this article are solely those of the authors and do not necessarily represent those of their affiliated organizations, or those of the publisher, the editors and the reviewers. Any product that may be evaluated in this article, or claim that may be made by its manufacturer, is not guaranteed or endorsed by the publisher.

## Footnotes

1 In the below paragraph we have included the prevalence rates in parenthesis, and marked with ^∗^ variables found in the sample at focus in the present study.

2
https://www.gu.se/en/research/the-octo-twin-study-origins-of-variance-in-the-old-old
